# Underrated primary biogenic origin and lifetime of atmospheric formic and acetic acid

**DOI:** 10.1038/s41598-021-86542-2

**Published:** 2021-03-30

**Authors:** Xinqing Lee, Daikuan Huang, Qi Liu, Xueyan Liu, Hui Zhou, Qian Wang, Yuena Ma

**Affiliations:** 1 State Key Laboratory of Environmental Geochemistry, Institute of Geochemistry, Chinese Academy of Sciences, 99 Lincheng West Road, Guiyang, 550081 Guizhou China; 2 State Key Laboratory of Ore Deposit Geochemistry, Institute of Geochemistry, Chinese Academy of Sciences, Guiyang, 550081 Guizhou China; 3 School of Earth System Science, Tianjin University, Tianjin, 300072 China; 4 Present Address: Guizhou Environmental Science Research and Design Institute, 217 Qianling Road, Guiyang, 550081 Guizhou China

**Keywords:** Carbon cycle, Atmospheric chemistry

## Abstract

Formic and acetic acids are ubiquitous in the troposphere, playing an important role in the atmospheric chemistry. Recent model studies ended up with substantial low bias on their tropospheric budgets presumably due to a large missing biogenic source derived most likely from photochemical oxidation of long-lived volatile organic compound(s), i.e., a secondary biogenic emission. Here, by studying the stable carbon isotope composition of formic and acetic acid in couple in the troposphere and relevant sources, we find the gap relates to primary biogenic emission and atmospheric lifetime of the acids. We show the primary biogenic emission is only second to the secondary biogenic emission as a strong source. Marine emission is the least one yet present in all the tropospheric environments except some local air. Long-distance transport of this origin indicates the lifetime over 5 days for both acids. Our results indicate that recent simulations underrated both primary biogenic emission and the lifetime. These underestimations would inevitably bias low the modeled results, especially in the low and free troposphere where primary biogenic emission and lifetime has the most pronounced influence, respectively.

## Introduction

As organic acids, formic and acetic acid originate primarily from biosphere^1–3^, forest in particular^4–7^. Being the major component of the biosphere, forest produces formic and acetic acid directly in plant growth^8–10^, dominating the source of primary biogenic emission^11^. It also generates a variety of non-methane volatile organic compounds(VOCs), mostly isoprene^12^, a short-lived and most abundant^13^ VOC emitted primarily by trees during day time^14,15^. These VOCs give rise to formic and acetic acid in subsequent photochemical oxidation^16–18^, constituting the most of secondary biogenic emission^19,20^. Other sources observed include fossil fuel combustion^21,22^, biomass burning^23,24^, soil respiration^25^, marine release^2,26,27^, ant emission^28^, and plastic and food production^29^. These sources, however, are generally weaker than the biogenic ones^30,31^. Based on the dominance of the biogenic origins, budgets of the organic acids in the troposphere were simulated recently to infer their biogeochemical cycles, as well as the interaction between biosphere and atmosphere^32–37^. The results, however, are substantially lower than observations with significant bias occurring in the low troposphere^35^, the boundary layer in particular^35,38^, as well as in the free troposphere^38^, the mid-latitude of northern hemisphere and the northern polar region^35^. The gap was presumably caused by a missing biogenic source^5,38^, particularly in the form of photochemical oxidation of long-lived VOC precursor(s)^35^, i.e., a secondary biogenic emission. Such a large source, however, was not found despite a number of subsequent researches^20,39–44^. By studying the stable carbon isotope ratio (^13^C/^12^C ) of formic and acetic acid in couple in the troposphere and relevant sources, we find the primary biogenic emission is much more important and the atmospheric lifetime of the acids much longer in comparison to the model studies. Their underestimations would inevitably lead to the low bias on the acids’ tropospheric budgets.

## Brief description of the methods

We report the ^13^C/^12^C as δ^13^C, which is per mille deviation to the value of international standard Vienna Pee Dee Bolemnite (VPDB), following the equation:

$$\updelta^{{{13}}} {\text{C }} = \, \left( {\left( {^{{{13}}} {\text{C}}/^{{{12}}} {\text{C}}_{{}} } \right)_{{{\text{sample}}}} /\left( {^{{{13}}} {\text{C}}/^{{{12}}} {\text{C}}} \right)_{{{\text{standard}}}} - {1}} \right)*{1}000$$

To determine δ^13^C of formic and acetic acid in the troposphere, we analyzed both air and precipitation (Table 1). The air samples were collected at two kinds of environments in Guiyang, the capital city of Guizhou province in inland southwest China. One is over a traffic cross in the downtown street valley (sample C), the other above the canopy of a small forest within the metropolis (sample D). The latter is also the site for collection of precipitation samples (sample 1–6). We also used data published in literatures that analyzed both formic and acetic acid. These analyses involve air from urban to rural environments, as well as precipitation in semi-remote region^45^.

**Table 1:** Tropospheric environments and sources analyzed in this study.

Air				Precipitation			
Sample	Date	Environ. or source	N	Sample	Date	Rain(mm)	N
A	Aug. 20	Fossil fuel combustion	3	1	Aug. 25–26	42.7	6
B	Aug. 30	Suburban forest	4	2	Sep. 02–03	52.9	6
C	Aug. 28	Downtown street valley	6	3	Sep. 13–14	17.5	6
D	Aug. 27	Urban forest	6	4	Sep. 24–25	1.4	6
	Aug. 31	Urban forest	7	5	Oct. 03–04	49.7	6
	Sep. 01	Urban forest	3	6	Oct. 08–13	9.0	6

Sample A and B stand for the source of anthropogenic air pollution and primary biogenic emission, respectively, all the rest samples for the tropospheric environments. N indicates the number of samples collected and measured.

The sources we studied include the primary biogenic emission, secondary biogenic emission, fossil fuel combustion and marine release. These sources are continuous in the emission and thus most likely to have a broad atmospheric influence. Biomass burning, soil respiration, industrial productions and ant release were not taken into account because they are either sporadic in occurrence, or trivial in importance, or local in influence, or producing only a single acid.

We obtained the δ^13^C of fossil fuel combustion by analyzing pipe exhaust of an idle Toyota Land Cruiser (sample A), and the δ^13^C of primary biogenic emission by analyzing air on the ground level in the suburban forest zone of Guiyang (sample B). We estimated the δ^13^C of secondary biogenic emission based on the average δ^13^C of C_3_ plants, which dominate forests worldwide^46^, and the reported isotope fractionation in producing isoprene, as well as on the intermolecular isotope fractionation between formic and acetic acid, which we measured experimentally on isoprene photochemical oxidation. Other VOCs, such as aldehydes and other alkenes, also generate the acids in the oxidation, their production is nevertheless relatively small and uncertain^29,47^. The detailed sampling methods and analytical procedures as well as the estimation of the δ^13^C are presented in the section of “Detailed methods” at the end of this paper.

Besides the sources measured experimentally in this study, we also inferred the δ^13^C of marine origin from the reported data of precipitation in Los Angeles, USA^48^, where the rainfall developed primarily from the moisture of the Eastern Pacific^49,50^.

## Results

**Figure. 1:**
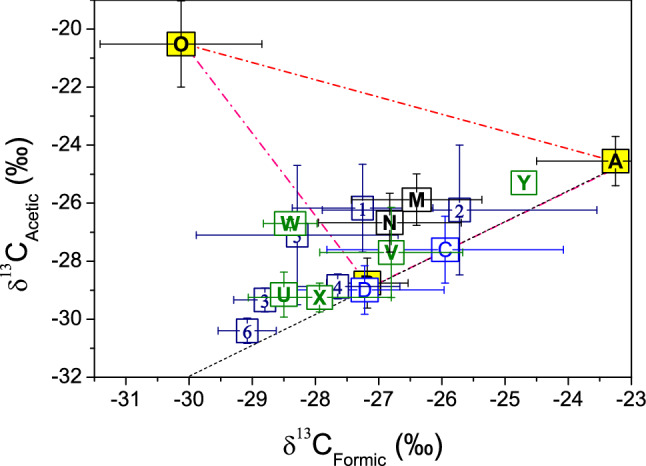
Mean δ^13^C of formic and acetic acid in the tropospheric environments and the sources of fossil fuel combustion, marine release and primary biogenic emission. The yellow-colored squares indicate the sources. A: fossil fuel combustion; B (covered mostly by D): primary biogenic emission; O: marine emission as averaged from the δ^13^C of precipitation at Westwood, Los Angeles, California, USA^48^; C: air in the street valley of downtown Guiyang; D: air over the urban forest in Guiyang; M and N: urban air during March and August–September, respectively, in Zurich, Switzerland^51^; U: urban air at Oslo, Norway; V: rural air at Tommerup, Denmark; W and X: air at semi-remote Ulfborg and Anholt, Denmark, respectively; Y: precipitation at semi-remote Anholt, Denmark^45^; digits 1–6: precipitation events in Guiyang as specified in Table 1. Error bars are 2σ standard deviation. The dash-dot lines link the source, the dash line indicates δ^13^C_Formic_/δ^13^C_Acetic_ of 0.94.

We show the sources of fossil fuel combustion, primary biogenic emission and marine release are separated distinctively in the form of a triangle in the isotope system of formic and acetic acid (Fig. 1), and the former two sources has the same δ^13^C_Formic_/δ^13^C_Acetic_ as 0.94. The tropospheric environments we measured in Guiyang as well as those reported by others in Switzerland^51^, Norway and Denmark^45^ all display a δ^13^C_Formic_/δ^13^C_Acetic_ above the line of 0.94 except the air in the street valley and over the urban forest in Guiyang, which are right on the line. The δ^13^C of the troposphere distribute only partly within the triangle of the three sources, those outside the enclosure demand one more source that is supposedly more depleted in ^13^C in both formic and acetic acid. This source is proved to be the secondary biogenic emission. As indicated by our experimental results on isoprene photochemical oxidation, the fractionation factor between formic and acetic acid averages 1.002 (Table S1), and the estimated δ^13^C is − 29.4‰ and − 31.4‰ for the secondary formic and acetic acid, respectively, with δ^13^C_Formic_/δ^13^C_Acetic_ about 0.94 as well. These results locate the source of the secondary biogenic emission in line with fossil fuel combustion and primary biogenic emission in terms of δ^13^C_Formic_/δ^13^C_Acetic_ but to their lower left in the isotopic compositions. The photochemical experiments further showed that the δ^13^C_isoprene_ calculated from the measured δ^13^C_Formic_ and δ^13^C_Acetic_ differs to the true δ^13^C_isoprene_ by only 0.1‰ (Table S1), the little difference confirms that the carbon in isoprene is transferred almost completely into the organic acids.

**Figure. 2:**
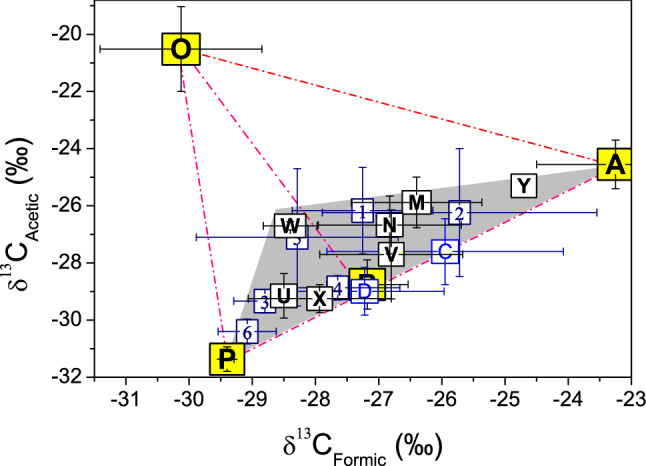
Mean δ^13^C of all the sources and tropospheric environments studied. P: the source of secondary biogenic emission. The rest dots and lines are the same as in Fig. 1. The gray triangle indicates the range of the δ^13^C in the troposphere.

Addition of the source of secondary biogenic emission makes all the δ^13^C of the tropospheric environments well within enclosure of the sources (Fig. 2), indicating that we captured the major sources of the tropospheric acids in this study. The precipitation at semi-remote Anholt, Denmark, was noted with anthropogenic pollution^45^, it is closest to the fossil fuel combustion in the isotopic system, confirming the high contribution of anthropogenic source. Anholt and Tommerup, Denmark, as well as Oslo, Norway, are all situated in the lee of continent in relation to the prevailing wind, i.e., the westerly that blows eastwards from the North Atlantic, despite their difference in such environment as the semi-remote, rural and urban, respectively^45^. The air was subject to terrestrial (biogenic and anthropogenic) influence before reaching to these places. Ulfborg, Denmark, on the other hand, is located at the west coast of the country, facing the North Sea and the prevailing wind thus with minimum terrestrial influence. As a result, Ulfborg is much closer to the marine source than Anholt, Tommerup and Oslo in the isotopic system, manifesting its higher proportion of the marine acids. In fact, it is also the closest or highest among all the tropospheric environments in question thanks to its geographic and atmospheric situations. The air in Zurich, Switzerland, was affected by stronger photo-oxidation in August–September than in March^51^, the former is closer to the source of secondary biogenic emission than the latter in the isotope compositions, corroborating that the air of summer season has higher acids of secondary biogenic origin. These consistencies indicate that the isotopic compositions of the double acids point to their origins very well in the troposphere.

The δ^13^C of troposphere varies substantially between samplings even at the same site as indicated by the large standard deviation of the means. This is consistent with the volatile nature of atmosphere. Nevertheless, all the data are concentrated in an area that fits well a triangle as grayed in the isotopic system (Fig. 2). The distribution is away from the marine source while inclining toward the secondary biogenic emission. It indicates that the oceanic contribution is the least, especially in terms of acetic acid, while the secondary biogenic one most important to the tropospheric acids. This is in agreement with recent model studies^31,35,52^ and also confirmed by the model of Stable Isotope Analysis in R (SIAR) (Table S2), which predicts contribution of each source to the mixture based on probability distribution^53^. It shows that, on average, the marine origin accounts for 16% while the secondary biogenic emission 34% of acetic acid in the troposphere. The vehicle exhaust contributes 21% following the marine release as the second least source. The primary biogenic emission is located near the mid-point in the bottom of the gray triangle, close to most tropospheric environments, indicating its high contribution to the acids in these environments. As proved by SIAR, the average contribution is 29% in case of acetic acid, only second to the secondary biogenic emission. The air over the urban forest in Guiyang is supposedly subject to higher anthropogenic pollution than the suburban forest. Nevertheless, their δ^13^C differ only by 0.04‰ and 0.24‰ in formic and acetic acid, respectively. These minute differences suggest that the primary biogenic emission is so strong that it overwhelms the influence of anthropogenic pollution. The δ^13^C in both forests are distinct from those of secondary biogenic emission, ruling out the possibility of the secondary emission as the major source in/over the forest.

All the precipitation at Guiyang has the acids of marine origin despite the long distance to the oceans. Back trajectory analyses on the large precipitation events, i.e., 1, 2, 3 and 5, which formed by marine airmass meeting the continental one^54^, show that both formic and acetic acid survived the 120 h of transportation (Fig. S2). This indicates that the tropospheric lifetime of both acids is 5 days at least. The air over the urban forest as well as in the downtown street valley of Guiyang is free of the oceanic acids, suggesting that both environments have been closed from outside exchange for longer-than-the-lifetime period.

## Discussion

The contribution of the primary biogenic emission projected by SIAR is 10 times as high as that predicted in recent simulation studies^35^. Although the SIAR prediction is only a statistical probability, the substantial difference still makes us believe that the simulations significantly undervalued the source of primary biogenic emission. Should this be true, significant low bias of the modeled budget would occur in situations with strong primary biogenic emission. This is exactly the case with the modeled results, which biased lowest in the low troposphere^35,38^ due to its proximity to the primary biogenic source, especially in the mid-latitude of the northern hemisphere, where terrestrial area amounts the largest on the globe and thus the primary biogenic emission has the strongest influence.

The lifetime of atmospheric formic acid is reported as 3.2 days or even as long as 4.5 days in recent model studies^5,35^. Compared to the lifetime revealed by the long-distance transport in this study, these were underestimated it by 56% and 11% at least. The modeled lifetime of acetic acid is 2.3 days^35^, which is undervalued by 117% at least. These underestimations certainly bias low the modeled tropospheric budget of the acids, thus demanding even larger emission flux of sources to reconcile the modeled budgets with observations. In places like the free troposphere and polar region, however, it is still unable to fill the gap even after substantially increasing the strength of the major sources including the primary biogenic emission^35,38^. The extended tropospheric lifetime of the acids provides an insight into this enigma.

## Conclusions

Our results show that the primary biogenic emission is the second largest source of formic and acetic acid in the troposphere following the secondary biogenic emission, i.e., the photo-oxidation of VOC precursors. Therefore, the simulation studies underestimated the importance of this source. We also show that the lifetime of the acids in the troposphere exceeds 5 days, which is also longer than those in recent model studies. Based on these findings, we propose that these underestimations are relevant to the low bias of the simulated tropospheric budget of the acids. These results were obtained independently of the previous approaches used, thus shedding new light into the critical issues on the biogeochemical cycle of the acids.

## Detailed methods

### Sampling sites in field observations

Guiyang is a mountainous city in the subtropical southwest China (N26.57, E106.71). It is home to 2.5 million people with small forested hills dotting the downtown area. As one of the hills, Guanfeng Hill is 42 m high and occupies an area about 1000 m^2^. 10 km to the east of the metropolis, there lies a zone of suburban forest about 3 km wide and 30 km long, extending roughly in the south-north direction.

### Sampling in the field

We sampled air on the ground level in the center of the suburban forest (Sample D in Table 1) and over the forest canopy by the edge of Guanfeng Hill (Sample B), as well as on a 5 m-tall pedestrian bridge over a downtown street-cross with heavy traffics at the center of the metropolis (Sample C). We also collected precipitation samples over the forest canopy at Guanfeng Hill (Sample 1–6). These samples were collected on the event basis using an auto-sampler (APS-2B, Changsha Company, China). After the collection, we stored the precipitation samples at − 18 °C if not processed immediately^55^. We sampled the air of fossil fuel combustion in front of the exhaust pipe of an idle Toyota Land Cruiser with an odometer about 100 thousand kilometers (Sample A).

### Extraction of formic and acetic acid

Formic and acetic acids in the air were sampled by a dynamic solid-phase micro-extraction device, the NeedlEx, the type of fatty acids (Shinwa Chemical Industries, Ltd, Japan). It is a needle filled by adsorbent with affinity to fatty acids. To prevent possible clog of the needle, we attached a glass fiber filter (1.2 μm) on the head during the extraction. Due to the usually low concentration of the organic acids in the air, 3L of the air were drawn through the NeedlEx by a sucking pump. The organic acids were trapped by the adsorbent as the air passes through.

To sample the acids in precipitation, we concentrated the acids prior to the extraction due to the low concentrations in most of the samples (usually about a few μmol/L). To do this, we first neutralized 1L of the sample to pH 7 using 6 mol/L of NaOH solution, and then loaded it into 5 Supelclean LC-SAX SPE cartridges (Supelco, 500 mg/3 mL) hyphenated one after another by Teflon tubes. The cartridges retained the organic acids with other anions such as SO_4_^2−^, NO_3_^−^ and Cl^−^ in the samples. We eluted the anions out by 20 mL of 2 mol/L HCl and adjusted the pH of the eluted solution to 2.3 using 4 mol/L of H_3_PO_4_. After the preconcentration, we extracted the organic acids by the NeedlEx in a purge-and-trap way. Briefly, we transferred the eluent into a 40 mL vial and purged the solution with 1L Helium gas (99.999% purity) assisted by magnetic stirring at 2000 rpm. The headspace gas was sucking through the NeedlEx for extraction of the organic acids. The detailed setup of the purge-and-trap system is available in our previous publication^56^.

### Measurement of the isotope ratio

We introduced the loaded NeedlEx directly into the injection port of a HP6890N Gas Chromatography (GC), which was installed with a Stabilwax-DA fused-silica capillary column (30 m long, 0.32 mm i.d., 0.25 lm df, Restek, Bellefonte, PA, USA), and hyphenated via a combustion interface with an Isotope Ratio Mass Spectrometry (IRMS) (Micromass Isoprime). By thermo-desorption assisted with flush of 1μL Helium gas, the organic acids trapped in the NeedlEx were delivered into the instrument for subsequent separation, oxidation and determination of ^13^C/^12^C. The entire processes incurred no isotope fractionation and the analytical precision is better than 0.9‰ for both acids at concentrations above 1 mg/L^56^.

### Experiment on isoprene photo-oxidation

Our experiment is analogous to that of Paulot et al. (2009)^19^. We used Teflon FEP gas bags (GSBTeco, FEP31C-2PP-15L) as the reaction chamber. We first filled it with 14L of N_2_ gas (Local supplier, 99.99%), then added by a micro-syringe with 7μL of NO gas (Summit, 99.9%), 1.5μL of the headspace gas of isoprene (Sigma-Aldrich, 99%) and 30μL of the headspace gas of H_2_O_2_ (Sigma-Aldrich, 34.5–36.5%). We exposed the filled gas bag to sunlight from 9 AM to 7 PM for photochemical reactions while blowing it with ambient air to drop the temperature. After 16 h of reaction, we extracted the produced formic and acetic acid for the isotopic analysis following the procedures of air extraction described above. The results were used to calculate the intermolecular isotope fractionation factor (α) following the equation:

$$\upalpha _{{{\text{formic}} - {\text{acetic}}}} = \, (\updelta ^{{{13}}} {\text{C}}_{{{\text{formic}}}} + {1}000)/(\updelta ^{{{13}}} {\text{C}}_{{{\text{acetic}}}} + {1}000)$$

### Estimation of δ^13^C of secondary biogenic emission

δ^13^C of the plant in C_3_ photosynthetic pathway ranges from − 32 to − 22‰ with a mean value − 27‰^57^. The plant synthesizes isoprene discriminating against ^13^C by 2.8‰ on average^58,59^. Accordingly, we estimated the average δ^13^C of the biogenic isoprene as − 29.8‰. Photo-oxidation of isoprene generates more formic than acetic acid^19^ with an average ratio of formic/acetic as 3.9 (Fig. S1). Assuming that the final products of the photo-oxidation are primarily formic and acetic acid, which is justified by experimental data (Table S1), we are able to establish the following equations:

1 $${\text{x}} + {\text{y }} = {1}$$

2 $${\text{x}}/{\text{y}} = {3}.{9}$$

3 $${\text{x}} \cdot\updelta ^{{{13}}} {\text{C}}_{{{\text{Formic}}}} + {\text{ y}} \cdot\updelta ^{{{13}}} {\text{C}}_{{{\text{Acetic}}}} = \,\updelta ^{{{13}}} {\text{C}}_{{{\text{Isoprene}}}}$$

4 $$\updelta ^{{{13}}} {\text{C}}_{{{\text{Formic}}}} {-} \,\updelta ^{{{13}}} {\text{C}}_{{{\text{Acetic}}}} = { 1}0^{{3}} \cdot {\text{Ln}}(\upalpha _{{{\text{formic}} - {\text{acetic}}}} )$$

where x and y denote the percent productivity of formic and acetic acid, respectively.

Solving the group of equations, we obtained δ^13^C of formic and acetic acid originated from photochemical oxidation of the biogenic isoprene.

### Back trajectory analysis

We analyzed the back trajectory of the airmass movements by the protocol of Draxler and Rolph^60^.

### Calculation of the sources contribution to tropospheric acetic acid

We assume that acetic acid in the atmosphere was derived from the four major sources: marine release (S1), fossil fuel combustion (S2), primary biogenic emission (S3), and secondary biogenic emission (S4). Based on stable isotope mass-balance theory, δ^13^C signatures of the mixture are determined by δ^13^C values and fractional contributions of each source to the mixture. Accordingly, we have the equations as following:

$$\updelta ^{{{13}}} {\text{C}}_{{{\text{Acetic}}}} = F_{{{\text{S1}}}} \times \,\updelta ^{{{13}}} {\text{C}}_{{{\text{S1}}}} + F_{{{\text{S2}}}} \times \,\updelta ^{{{13}}} {\text{C}}_{{{\text{S2}}}} + F_{{{\text{S3}}}} \times \,\updelta ^{{{13}}} {\text{C}}_{{{\text{S3}}}} + F_{{{\text{S4}}}} \times \,\updelta ^{{{13}}} {\text{C}}_{{{\text{S4}}}}$$

where δ^13^C_Acetic_ is δ^13^C values of acetic acid in the troposphere. *F*_S1_, *F*_S2_, *F*_S3_, and *F*_S4_ the fractional contributions of the sources S1, S2, S3 and S4 to the mixed acetic acid in the troposphere, respectively. *F*_S1_ + *F*_S2_ + *F*_S3_ + *F*_S4_ = 1. δ^13^C_S1_, δ^13^C_S2_, δ^13^C_S3_, and δ^13^C_S4_ denote δ^13^C values of acetic acid from S1, S2, S3, and S4, respectively.

*F*_S1_, *F*_S2_, *F*_S3_, and *F*_S4_ were calculated using the Stable Isotope Analysis in R (the SIAR model: http://cran-project.org/web/packages/siar/index.html). The SD values of each *F* value in each run were calculated SD values of the 10,000 contribution data output from the SIAR model. Uncertainties of mean *F* values can be propagated by the Monte Carlo method (MCM) as the SD values of corresponding *F* values of different sample replicates.

## Supplementary Information

Supplementary Information
