# Supplementary material for: Underrated primary biogenic origin and lifetime of atmospheric formic and acetic acid
Source: Sci Rep. 2021 Mar 30;11:7176. doi: 10.1038/s41598-021-86542-2 (PMC8009910; doi:10.1038/s41598-021-86542-2)
Supplement: Supplementary file 1 — Supplementary Information [file 41598_2021_86542_MOESM1_ESM.pdf]

SUPPLEMENTARY MATERIAL

Underrated primary biogenic origin and lifetime of atmospheric formic and acetic acid

Xinqing Lee[1*], Daikuan Huang[1], Qi Liu[2], Xueyan Liu[3], Hui Zhou[1], Qian Wang[1], Yuena Ma[1]

[1.] State Key Laboratory of Environmental Geochemistry, Institute of Geochemistry, Chinese Academy of Sciences, Guiyang 550081, Guizhou, China

[2.] State Key Laboratory of Ore Deposit Geochemistry, Institute of Geochemistry, Chinese Academy of Sciences, Guiyang 550081, Guizhou, China

[3.] School of Earth System Science, Tianjin University, Tianjin 300072, China

[*] To whom correspondence should be addressed. email： lee@mail.gyig.ac.cn; address: 99 Lincheng West Road, Guiyang 550081, Guizhou, China

1. Ratio of formic acid vs. acetic acid produced in the photo-oxidation of isoprene

As shown by the experiment of Paulot et al. (2009) [1], formic and acetic acid are the most abundant products and also the ever increasing ones in photochemical oxidation of isoprene (Supplementary Fig. 1) while the other carbon-bearing products decreased quickly after peaking at a short time. It suggests that the acids are the final products while the others intermediates. Given longer reaction time as occurred in the troposphere, it is justifiable to assume the carbon of isoprene would be transferred predominantly into the organic acids. This provided the basis for equation 3 in our estimation of $\delta^{13}C$ of the acids from isoprene photo-oxidation (See Detailed methods).

The data of Paulot et al. (2009) [1] showed that production of formic acid is higher than that of acetic in the oxidation of isoprene. The ratio of formic/acetic is not constant but changes with time with a mean value 3.9. We take the mean value as the production ratio in equation 2 in the estimation of $\delta^{13}C$ of the acids from isoprene photo-oxidation (See Detailed methods).

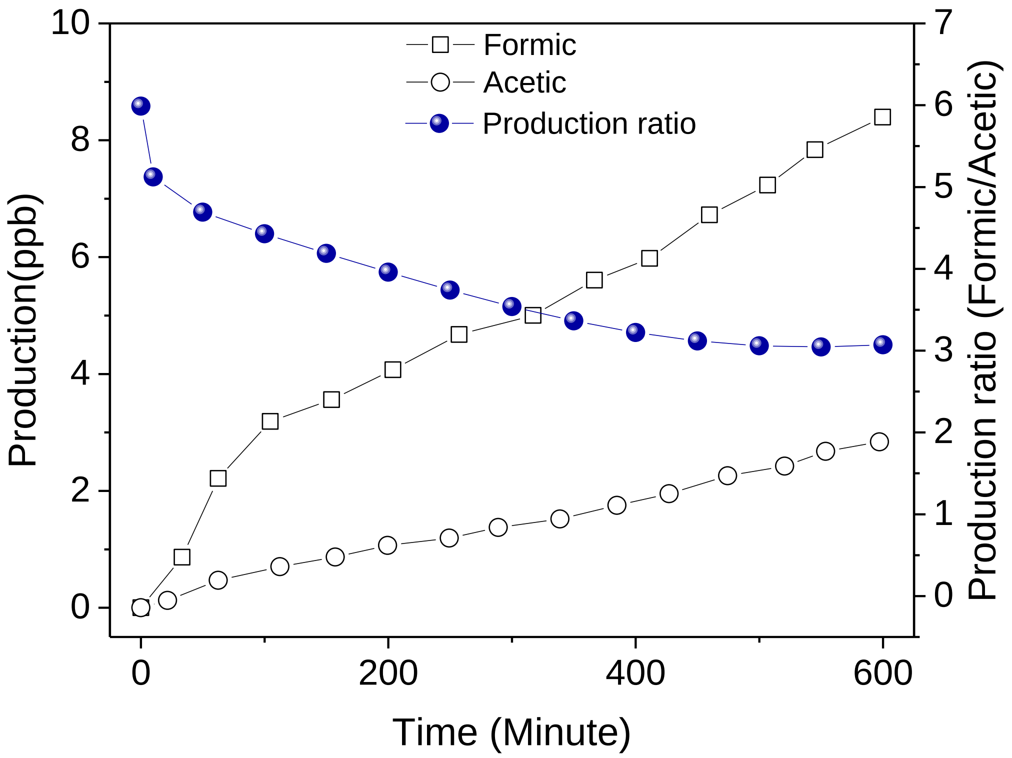

Supplementary Fig. 1. Production of formic and acetic acid and their ratio in the photo-oxidation of isoprene. The production data are digitalized from the curves of Fig. 14 and 15 of Paulot et al., (2009)[1] and those of formic/acetic from the fitting function of formic acid divided by that of acetic acid.

2. Isotopic fractionation between formic and acetic acid generated by isoprene oxidation

To acquire the carbon isotope fractionation factor (α) between formic and acetic acid, we did photochemical oxidation experiments using the reagent of isoprene purchased from Sigma-Aldrich. The experiments enabled us to measure $\delta^{13}C$ of formic and acetic acid produced from isoprene oxidation, i.e., $\delta^{13}C_{formic}$ and $\delta^{13}C_{acetic}$ in Supplementary Table 1. Based on these isotopic compostions, we calculated the isotope fractionation factor α. The results show the average α is 1.002(Supplementary Table 1). The calculated $\delta^{13}C_{isoprene}$ is close to the calibrated one, i.e., the true value, confirming that the carbon in isoprene is transferred almost completely into the organic acids[1].

Supplementary Table 1. The carbon isotope fractionation between formic and acetic acid produced in the photochemical oxidation of isoprene.

Experiments	$\delta^{13}C_{isoprene}$*	$\delta^{13}C_{formic}$	$\delta^{13}C_{acetic}$	$\delta^{13}C_{isoprene}$**	$\alpha_{formic\text{-}acetic}$
1	-23.1	-20.8	-23.4	-21.3	1.003
2	-23.1	-22.7	-24.0	-23.0	1.001
3	-23.1	-23.3	-25.1	-23.7	1.002
4	-23.1	-21.5	-23.9	-22.0	1.002
5	-23.1	-24.0	-26.1	-24.4	1.002
6	-23.1	-22.7	-24.1	-23.0	1.001
Mean	-23.1	-22.5	-24.4	-23.0	1.002

*$\delta^{13}C$ of the purchased isoprene reagent as calibrated by complete combustion and dual inlet mass spectrometry measurement.

**$\delta^{13}C$ of isoprene calculated from the measured $\delta^{13}C_{formic}$ and $\delta^{13}C_{acetic}$ by equation 3.

3. SIAR-estimated contributions of the sources to the mixture

Supplementary Table 2. Contribution of the sources to the tropospheric acetic acid as estimated by SIAR.

	Marine source		Fuel combustion		Primary biogenic		Secondary biogenic	
Environments	Mean(%)	1σ	Mean(%)	1σ	Mean(%)	1σ	Mean(%)	1σ
Precipitation 1	22	11	25	14	26	14	27	12
Precipitation 2	14	10	22	13	30	15	34	13
Precipitation 3	19	11	24	13	27	14	30	13
Precipitation 4	11	8	20	12	31	16	38	13
Precipitation 5	12	9	21	12	31	16	36	13
Precipitation 6	16	10	23	13	29	15	32	12
Urban forest-1	6	5	11	9	30	16	53	15
Urban forest-2	9	6	15	11	33	17	43	13
Urban forest-3	15	11	22	13	29	15	34	13
Downtown street	14	9	24	13	30	15	32	12
Zurich-summer	22	9	24	14	27	14	27	11
Zurich-winter	28	9	25	14	24	13	23	11
Norway air	17	10	23	13	28	14	31	12
**Mean**	**16**		**21**		**29**		**34**	

4. Results of back trajectory analysis of the airmass movements for the precipitation events

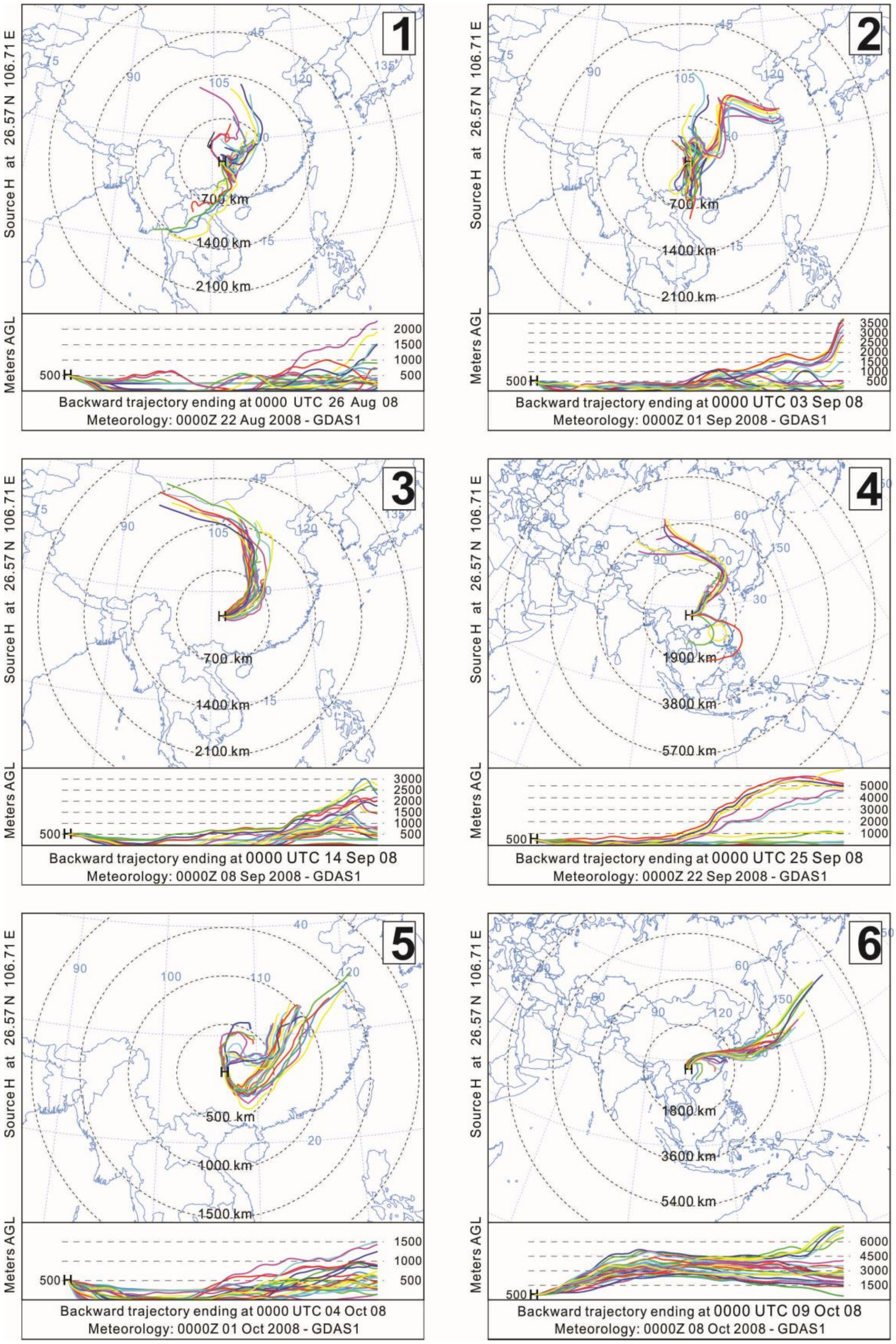

Supplementary Fig. 2. Back trajectory of the airmass movements for the precipitation events

sampled in Guiyang. The number in the top-right of the panels indicates the precipitation event as in Table 1.

References

1. Paulot F, Crounse JD, Kjaergaard HG, Kroll JH, Seinfeld JH, Wennberg PO. Isoprene photooxidation: new insights into the production of acids and organic nitrates. *Atmos Chem Phys* 2009, **9**(4)**:** 1479-1501.
